# Orexin-A/Hypocretin-1 Controls the VTA-NAc Mesolimbic Pathway via Endocannabinoid-Mediated Disinhibition of Dopaminergic Neurons in Obese Mice

**DOI:** 10.3389/fnsyn.2021.622405

**Published:** 2021-02-04

**Authors:** Lea Tunisi, Livia D'Angelo, Alba Clara Fernández-Rilo, Nicola Forte, Fabiana Piscitelli, Roberta Imperatore, Paolo de Girolamo, Vincenzo Di Marzo, Luigia Cristino

**Affiliations:** ^1^Endocannabinoid Research Group, Institute of Biomolecular Chemistry, National Research Council, Pozzuoli, Italy; ^2^Department of Veterinary Medicine and Animal Productions, University of Naples Federico II, Naples, Italy; ^3^Canada Excellence Research Chair on the Microbiome-Endocannabinoidome Axis in Metabolic Health, Université Laval, Québec City, QC, Canada; ^4^Department of Sciences and Technologies, University of Sannio, Benevento, Italy; ^5^Heart and Lung Research Institute of Université Laval, and Institute for Nutrition and Functional Foods, Université Laval, Québec City, QC, Canada

**Keywords:** obesity, leptin, reward, orexin, cannabinoid receptor (CB1R), endocannabinoids

## Abstract

Disinhibition of orexin-A/hypocretin-1 (OX-A) release occurs to several output areas of the lateral hypothalamus (LH) in the brain of leptin knockout obese *ob/ob* mice. In this study, we have investigated whether a similar increase of OX-A release occurs to the ventral tegmental area (VTA), an orexinergic LH output area with functional effects on dopaminergic signaling at the mesolimbic circuit. By confocal and correlative light and electron microscopy (CLEM) morphological studies coupled to molecular, biochemical, and pharmacological approaches, we investigated OX-A-mediated dopaminergic signaling at the LH-VTA-nucleus accumbens (NAc) pathway in obese *ob/ob* mice compared to wild-type (wt) lean littermates. We found an elevation of OX-A trafficking and release to the VTA of *ob/ob* mice and consequent orexin receptor-1 (OX1R)-mediated over-activation of dopaminergic (DA) neurons via phospholipase C (PLC)/diacylglycerol lipase (DAGL-α)-induced biosynthesis of the endocannabinoid 2-arachidonoylglycerol (2-AG). In fact, by retrograde signaling to cannabinoid receptor type 1 (CB1R) at inhibitory inputs to DA neurons, 2-AG inhibited GABA release thus inducing an increase in DA concentration in the VTA and NAc of *ob/ob* mice. This effect was prevented by the OX1R antagonist SB-334867 (30 mg/Kg, i.p.), or the CB1R antagonist AM251 (10 mg/Kg, i.p.) and mimicked by OX-A injection (40 μg/Kg, i.p.) in wt lean mice. Enhanced DA signaling to the NAc in *ob/ob* mice, or in OX-A-injected wt mice, was accompanied by β-arrestin2-mediated desensitization of dopamine D2 receptor (D2R) in a manner prevented by SB-334867 or the D2R antagonist L741 (1.5 mg/Kg, i.p.). These results further support the role of OX-A signaling in the control of neuroadaptive responses, such as compulsive reward-seeking behavior or binge-like consumption of high palatable food, and suggest that aberrant OX-A trafficking to the DA neurons in the VTA of *ob/ob* mice influences the D2R response at NAc, a main target area of the mesolimbic pathway, via 2-AG/CB1-mediated retrograde signaling.

**Graphical Abstract:**
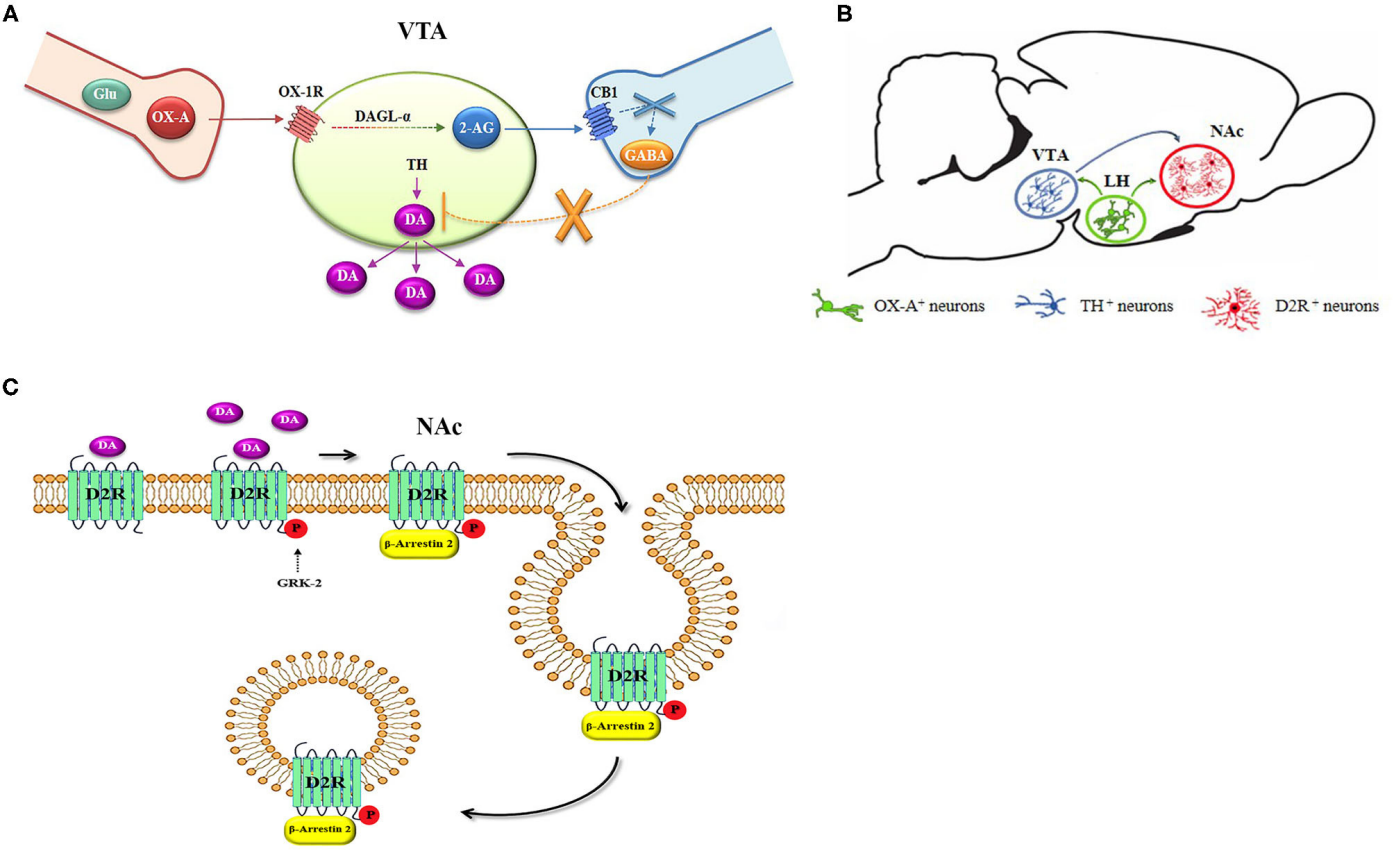
Scheme of the anatomical and functional pathway regulating the reinforcement of DA signaling in obese mice. **(A)** A Functional cross-talk between OX-A and 2-AG occurs in the DA neurons of the VTA. **(B)** OX-A and DA interplay at LH-VTA-NAc neuronal circuitry. **(C)** Scheme of the DA-mediated D2R desensitization by phosphorylation of G protein receptor kinase-2 (GRK-2) and β-arrestin2 signaling in the NAc, culminating in the internalization of the D2R receptor in endosomes.

## Introduction

Orexin-A/hypocretin-1 (hereafter referred to as OX-A) plays a prominent role in conditioned responses to stimuli associated with food and drug rewards by regulating the functional activity of DA neurons in the VTA (Harris et al., 2005; Tung et al., 2016). DA neurons are innervated by orexinergic fibers (Fadel and Deutch, 2002) and those projecting to the shell of NAc forming the mesolimbic pathway undergo an OX-mediated increase of firing rate (Baimel et al., 2017). Although both type 1 and 2 of orexin receptors, named OX1R and OX2R, are expressed in striatal regions, OX-A promotes consumption of highly palatable food mainly through OX1R activation (Harris et al., 2005; Borgland et al., 2006; Baimel and Borgland, 2015; Barson et al., 2015). Accordingly, i.p. or intracerebroventricular (i.c.v.) injection of OX-A increases food-intake in *ad-libitum* fed lean mice (Morello et al., 2016) whereas intra-VTA injection of OX-A increases intake of a diet with high contents of fat and sucrose solution in *ad-libitum* fed rats (Terrill et al., 2016), as well as a hedonic reaction to sucrose when injected in the rostromedial NAc (Castro et al., 2016). Moreover, OX-A injection in the paraventricular nucleus (PVN) of the thalamus increases the intake of a sucrose solution (Kay et al., 2014) and the release of DA in the NAc, thus promoting reward-related feeding in rats (Choi et al., 2012).

Acute consumption of an obesogenic high-fat diet (HFD) (Valdivia et al., 2014), or optogenetic stimulation of LH fibers projecting to DA neurons of the VTA (Nieh et al., 2015), promote a compulsive reward-seeking behavior inhibited by an OX1R antagonist (Piccoli et al., 2012). Accordingly, systemic injection of the OX1R antagonist SB-334867 reduces drinking of a sucrose solution (Terrill et al., 2016), acute intake of highly palatable food, and binge-like consumption of sucrose, together with reduced c-*Fos* activation in the VTA (White et al., 2005; Alcaraz-Iborra et al., 2014; Valdivia et al., 2014). Noteworthy, *Hcrtr1* knock-down rats undergo a reduction of both hedonic feeding and preference in the consumption of an HFD (Choi et al., 2012; Kay et al., 2014). Although both OX1R and OX2R are expressed in neurons of the mesolimbic regions, the neurobiological effects in reward-seeking behaviors are mainly due to OX-A binding to OX1R, whereas OX2R is more likely involved in sleep/wake cycle regulation and was suggested to participate in ethanol self-administration (Harris et al., 2005; Borgland et al., 2006; Barson et al., 2015; Baimel et al., 2017).

Endocannabinoids, especially 2-AG, have been implicated in food-seeking behaviors and obesity in a manner prevented by CB1R antagonists (Silvestri and Di Marzo, 2013). The endocannabinoid 2-AG is biosynthesized on demand to produce retrograde inhibition of neurotransmitter release at presynaptic CB1 receptors. We and others demonstrated that 2-AG can be generated when OX1R is activated by OX-A (Ho et al., 2011; Cristino et al., 2013). Activation of Gq by OX1R can trigger PCL/DAGL-α-mediated production and release of 2-AG from its diacylglycerol biosynthetic precursors (Imperatore et al., 2016; Chen et al., 2018). Furthermore, we have previously shown that obesity-associated changes occur at hypothalamic circuits in *ob/ob* mice because of the switch from predominantly excitatory to inhibitory CB1R-expressing inputs to OX-A neurons. These changes are accompanied by DSI-mediated disinhibition of OX-A neurons with consequent elevation of OX-A trafficking and release to many brain target areas including the periaqueductal gray (PAG) and arcuate nucleus (ARC) (Cristino et al., 2013, 2016; Morello et al., 2016; Becker et al., 2017).

Based on this background, we exploited the model of leptin knockout obese *ob/ob* mice to investigate if an enhancement of OX-A trafficking and release also occurs from the LH to the VTA. Obese *ob/ob* mice represent a good model to study the effect of aberrant OX-A signaling on dopamine trafficking in the mesolimbic pathway. This is of special relevance to the pivotal role of orexinergic, dopaminergic, and endocannabinoid signaling in the control of neuroadaptive responses underlying compulsive reward-seeking behaviors, which can contribute to obesity.

## Materials and Methods

### Animal and Drugs

The study has been performed according to the ARRIVE Guidelines to improve the reporting of bioscience research using laboratory animals. Experiments were performed in compliance with the European Union animal welfare guidelines [European Communities Council Directive of September 22, 2010 (2010/63/EU)] and the Italian Decree n.26/2014, authorization n. 152/2020-PR, Ministry of Health, Italy. Adult (16-wk-old) male C57BL/6j mice were purchased from Charles River Laboratories (Sulzfeld, Germany); male mice with spontaneous nonsense mutation of the *ob* gene for leptin (*ob/ob*, JAX mouse strain) B6.Cg-Lepob/J and WT *ob* gene expressing homozygous siblings of different ages were obtained from breeding *ob* gene heterozygotes and genotyped with PCR. Since orexin levels exhibit a diurnal fluctuation (concentrations increase during the dark period or active phase (i.e., ZT13- 24) and decrease during the light period or rest phase), the animals were maintained under a 12 h light:12 h dark cycle, light on at 8:00 PM, i.e., ZT0, for at least 4 weeks before euthanizing at ZT20-22. For the same reason, all the pharmacological treatments were performed at ZT20-22. Adult (16-wk-old) male B6.Cg-Tg(TH-GFP)21-31 (C57BL/6JJcl) mice were used for the electron microscopy analysis.

All mice were housed in controlled temperature and humidity conditions and fed *ad libitum* to exclude the effects of fasting on endocannabinoid-mediated plasticity in the VTA (Godfrey and Borgland, 2020). Wt and *ob/ob* mice were injected i.p. with several treatments as following: OX-A (Tocris, 40 μg/kg, 2 h), Leptin (Sigma Aldrich, 5 mg/kg, 2 h), SB-334867 (Tocris, 30 mg/kg for wt and 60 mg/kg for *ob/ob* mice, 3 h alone or 1 h before OX- A injection), AM251 (Tocris, 10 mg/kg, 3 h alone or 1 h before OX-A injection), O-7460 (Cayman, 12 mg/kg, 1 h alone or 30 min before OX-A injection), L741 (Tocris, 1.5 mg/kg, 30 min).

### Immunohistochemical Procedures

#### Single Antigen Immunohistochemistry

Animals were euthanized under isoflurane anesthesia, perfused transcardially with 0.9% saline and 4% paraformaldehyde/0.1 M phosphate buffer (PB), pH7.4. Brains were cryoprotected with 30% sucrose in PB and cut with Leica CM3050S cryostat into 10 μm-thick through the coronal plane. The sections were collected in three alternate series and maintained frozen until being processed.

For immunoperoxidase-labeling, the sections were pretreated with 0.75% H_2_O_2_ in an aqueous solution and then incubated overnight with primary antibody goat anti-Orexin-A (Santa Cruz Biotechnology, Inc., Dallas, Texas—USA) or mouse anti-tyrosine hydroxylase (TH) (Millipore), diluted 1:200 in TBS-T (tris-buffered saline solution (TBS), pH 7.3). Primary antibodies were revealed with appropriate biotin-conjugate secondary antibodies (diluted 1:100 in TBS-T; Vector Laboratories, Burlingame, CA) and avidin-biotin complex by using 3,3'-diaminobenzidine-4 (DAB) (Sigma Fast, Sigma-Aldrich, Louis, MO—U.S.A.) as chromogen substrate. The immunodensity of OX-A or TH immunoreactivity in the VTA was evaluated by quantifying the optical density (O.D.) of immunoperoxidase signal from a region of interest (ROI) having a square-shaped area of 1 mm^2^ selected, per each hemisphere, through the entire VTA area from Bregma −2.92 to −3.88 mm by analyzing *n* ≥ 3 sections/mouse (*n* = 6 mice/genotype). Concerning the OX-A immunoreactivity, the O.D. value of the portion of tissue devoid of stained fibers in the ROI was measured and subtracted as background from the O.D. value of fibers of interest. Concerning the TH immunoreactivity, the O.D. was calculated from the single-immunoreactive cells included in the ROI and by selecting only those exhibiting the largest nucleus in the focal plane. All the O.D. quantifications were performed by using the LAS AF MetaMorph Imaging Software (Leica, Wetzlar, Germany) according to the formula: OD = log_10_ (255/I), where the “I” was the pixel intensity value given by the imaging software. All the analyzed sections were exposed to the same parameters of brightness, contrast and magnification.

#### Multiple Labeling With Immunofluorescence

Alternate brain slices (10 μm of thickness) containing dorsal or ventral striatum of mice were collected in three series of slides and then processed for multiple immunofluorescence by overnight incubation in a mixture of the following primary antibodies, each diluted 1:100 in PB-T: (1) goat anti-OX-A (Santa Cruz Biotechnology, Inc., Dallas, Texas–USA) and mouse anti-TH (Millipore); (2) mouse anti-TH (Millipore) and rabbit anti-Neuronal Marker (anti-NeuN) (Abcam); (3) goat anti-OX1R (Santa Cruz Biotechnology), guinea pig anti-diacylglycerol lipase-α (anti-DAGL-α) (kindly provided by Prof. Ken Mackie) and mouse anti-TH (Millipore); (4) goat anti-OX-A (Santa Cruz Biotechnology), guinea pig anti-vesicular glutamate transporter (anti-VGluT1) (Synaptic Systems) and mouse anti-TH (Millipore); (5) rabbit anti-CB1R antibody (anti C terminus 461-472, Abcam), guinea pig anti-vesicular GABA transporter (anti-VGAT) (Synaptic Systems) and mouse anti-TH (Millipore); (6) rabbit anti-dopamine D2 Receptor (anti-D2R) (Millipore) and goat anti-βarrestin2 (Santa Cruz Biotechnology); (7) goat anti c-*Fos* (Santa Cruz Biotechnology) and rabbit anti-D2R (Millipore).

After incubation with primary antibodies, the sections were treated with appropriate Alexa-488, −546, or −350 donkey anti-IgGs (Invitrogen LifeTechnology) secondary antibodies before being counterstained with nuclear dye DAPI (4′,6-diamidino-2-phenylindole). Controls of specificity of immunolabeling in multiple fluorescence experiments were performed by omission of primary and secondary antibodies or by preabsorption of primary antibodies with the respective blocking peptides.

Immunofluorescence was analyzed by the confocal microscopy Nikon Eclipse Ti2 and images acquired with the digital camera DS-Qi2 (Nikon) and processed by Image analysis software NIS-Elements C (Nikon, Florence, Italy). *N* = 6–10 z stacks were collected through each analyzed section every 0.5 μm throughout the area of interest to be processed by the imaging deconvolution software. For each section, the optical density zero value was assigned to the background (i.e., a tissue portion devoid of stained cell bodies or fibers). Unbiased stereological counting method based on the MetaMorph Imaging Software (Leica, Wetzlar, Germany) was applied for quantification of the relative abundance of TH-immunoreactive neurons by counting the number of TH/NeuN double immunoreactive neurons vs. the total number of NeuN-immunoreactive neurons quantified in 1 mm^2^ of a counting square-shaped frame selected, per each hemisphere, through the entire VTA area from Bregma −2.92 to −3.88 mm (*n* ≥ 3 sections/mouse; *n* = 6 mice/genotype). Quantification of the relative abundance of c-Fos/D2R double immunoreactive neurons was performed in 1 mm^2^ of a counting square-shaped frame selected, per each hemisphere, through the entire NAc area from Bregma +1.34 to +2.2 mm by analyzing *n* ≥ 3 sections/mouse (*n* = 6 mice/genotype).

### CLEM: OX1R and CB1R Immunoreactivity in Pre-embedding Electron Microscopy

Double pre-embedding immunogold labeling for observation at transmission electron microscopy (TEM) was performed in the VTA (50-μm-thick) of TH-eGFP fed *ad libitum* mice fixed with 3% paraformaldehyde/0.5–1% glutaraldehyde (vol/vol) in PB. The TH-eGFP neurons were selected by observation under appropriate epifluorescence microscopy by being easily recognizable at 488 nm excitation wavelength. The selected sections were incubated free-floating overnight at 4°C with the primary antibodies (rabbit anti-CB1R antibody, anti-C terminus 461–472, Abcam; and goat anti- OX1R, Santa Cruz), all diluted 1:100 in donkey serum blocking solution with 0.02% saponin. Subsequently, the sections were incubated in a mixture of 6 nm (for CB1R) and 10 nm (for OX1R) gold-conjugated secondary antibodies (Aurion), diluted 1:30 in donkey serum blocking solution with 0.02% saponin. Sections were treated with 0.5% OsO_4_ in PB for 30 min at 4°C, dehydrated in an ascending series of ethanol and propylene oxide, and embedded in TAAB 812 resin (TAAB). Ultrathin (50 nm thickness) sections were cut by vibratome (Leica), collected on Formvar-coated, single- or multiple-slot (50-mesh) grids, and stained with 0.65% lead citrate. Electron micrographs were taken with the TEM microscope (FEI Tecnai G2 Spirit TWIN). The TEM observation was limited to series sectioned up to 0.6–0.8 μm depth from the external surface of pre-embedded immunolabeled tissue. Additional sections were processed in parallel as controls of reaction by omitting both or one of the primary antibodies from the mixture. No labeling was detected in the control material.

### Lipid Extraction and 2-AG Measurement

VTA samples were dissected by micropunch of 1.5 mm diameter from each hemisphere and pooled for each mouse (*n* = 8 mice/genotype per group) and homogenized in 5 vol chloroform/methanol/Tris HCl 50 mM (2:1:1 by volume) containing 50 pmol of d5-2-arachidonoylglycerol (d5-2-AG) as internal standards. Homogenates were centrifuged at 13,000 × g for 16 min (4°C), the aqueous phase plus debris were collected and four times extracted with 1vol chloroform. The lipid-containing organic phases were dried and pre-purified by open-bed chromatography on silica columns eluted with increasing concentrations of methanol in chloroform. Fractions for 2-AG measurement were obtained by eluting the columns with 9:1 (by volume) chloroform/methanol and then analyzed by liquid chromatography atmospheric pressure chemical ionization-mass spectrometry (LC-APCI-MS).

### DA Measurement in VTA and NAc

DA levels were measured by using the DA enzyme-linked immune specific assay (ELISA) kit (Cusabio Biotech, Wuhan, China). According to the manufacturer's instructions, the tissue was rinsed with 1X PBS, homogenized in 1 ml of 1X PBS, and stored overnight at −20°C and, after two freeze-thaw cycles, the homogenates were centrifuged for 5 min at 5,000 × g in refrigerated condition (5°C). The absorbance was measured at 450 nm a wavelength by using the 96-well microplate spectrophotometer Multiskan GO (Thermo Scientific, Waltham, MA). DA quantification was performed in VTA or NAc samples dissected by micro punch of 1.5 mm diameter from each separate hemisphere of a mouse, *n* = 9 mice/genotype per group. Visceral tissue samples were used as controls to check the specificity of the assay and to avoid non-specific-binding and false-positive results.

### Co-immunoprecipitation Assay and Western Blotting

Punched micro-dissected Nac samples from both hemispheres were pooled for each mice (*n* = 8 mice/genotype) and protein extracts were homogenized in an appropriate ice-cold lysis buffer and the concentration was determined using the Lowry protein assay (#55000111, Bio-Rad). The co-immunoprecipitation was performed with the Dynabeads Protein G-Kit (Invitrogen Life Technology) according to the manufacturer's instructions. For western blot, the membranes were incubated overnight with rabbit anti-Dopamine D2 Receptor antibody (Millipore, catalog #AB5084P) or rabbit anti-β-Arrestin2 antibody (Cell Signaling Technology, catalog #3857) dissolved in TBS-BSA (4%) and then incubated for 1 h at room temperature with goat anti-rabbit IgG (H+L)-HRP conjugate antibody (Biorad, #1706515). The reactive bands of membranes were detected by chemiluminescence after a 5 min incubation without light with ECL (#170-5061, Bio-Rad) and visualized using Chemidoc MP Imaging System (#17001402, Bio-Rad). The images were analyzed and quantified using ImageJ software (imagej.nih.gov/ij/). The intensity of the bands of immunoblotted β-Arrestin2 protein was normalized with the D2R protein as the loading control.

### Statistical Analyses

Data are expressed as mean ± SEM and were analyzed with GraphPad Prism 6 software, version 6.05 (GraphPad, Inc.). Data are presented as a histogram with mean ± sem. D'Agostino-Pearson's normality test was used to confirm the normal distribution of the data. Statistical differences among the two groups were determined by a two-tail *t*-test with Welch's correction. When more than two groups were analyzed statistics were computed by one-way ANOVA followed by Bonferroni test or two-way ANOVA followed by *post hoc* Tukey tests for comparison among means. A level of confidence of *P* < 0.05 was employed for statistical significance.

## Results

### OX-A and TH Immunoreactivities Are Enhanced in the VTA of *ob/ob* Mice

**Figure 1:**
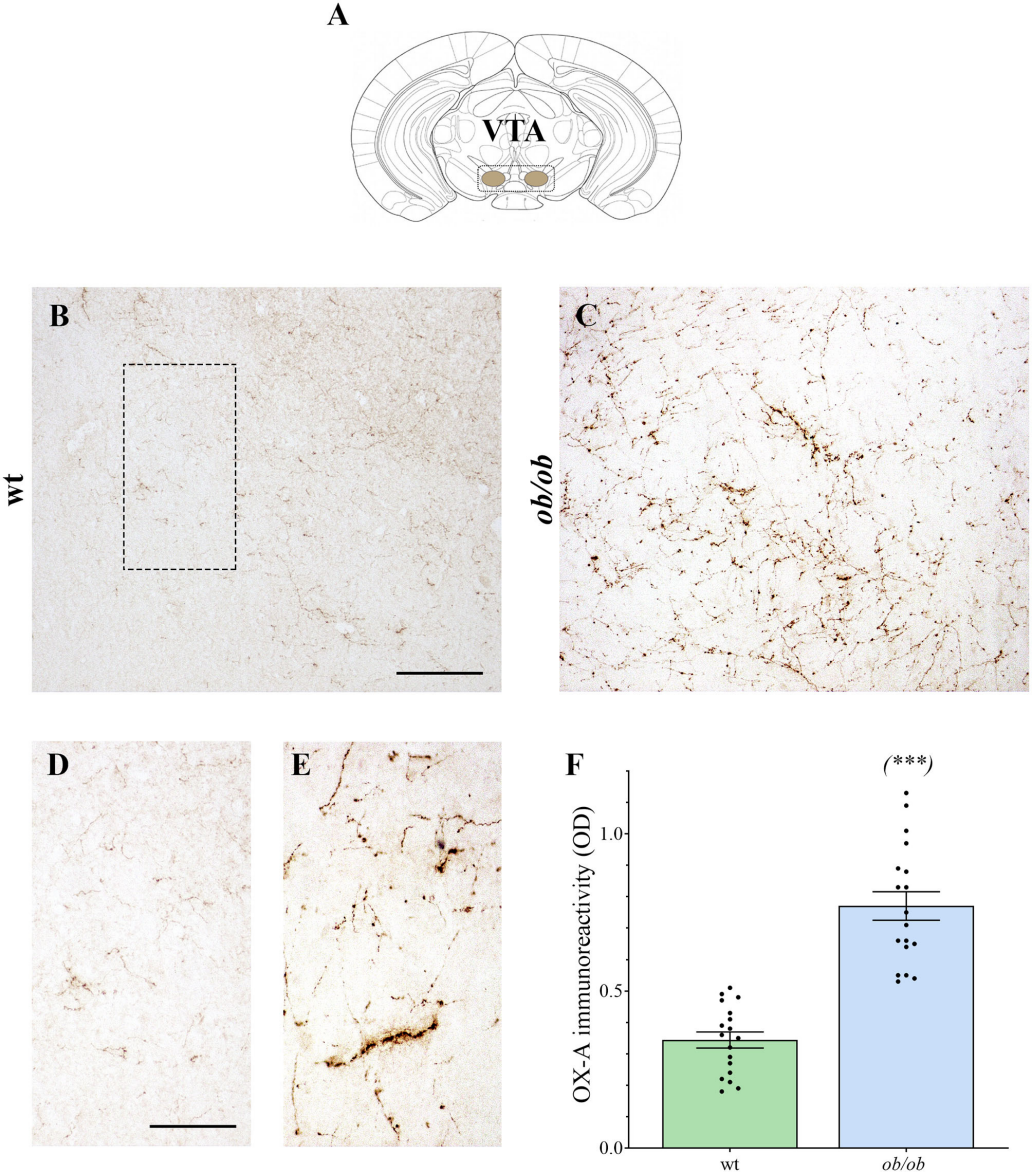
**(A)** Adaptation from the (Franklin and Paxinos, 1997) Mouse Brain Atlas, illustrating the VTA. **(B–E)** Representative peroxidase-based OX-A-immunoreactive staining in the VTA of wt and *ob/ob* mice showing a dense plexus of orexinergic fibers intensely immunoreactive in obese mice. Higher magnification of fields is depicted in **(D)** (wt) and **(E)** (*ob/ob*). Scale bar: 150 μm **(B,C)**, 50 μm **(D,E)**. **(F)** Bar graph of OX-A peroxidase-based optical density (OD). Data are means ± SEM from n ≥3 VTA sections/mouse, *n* = 6 mice/genotype; ***P < 0.001; two-tail *t*-test with Welch's correction.

**Figure 2:**
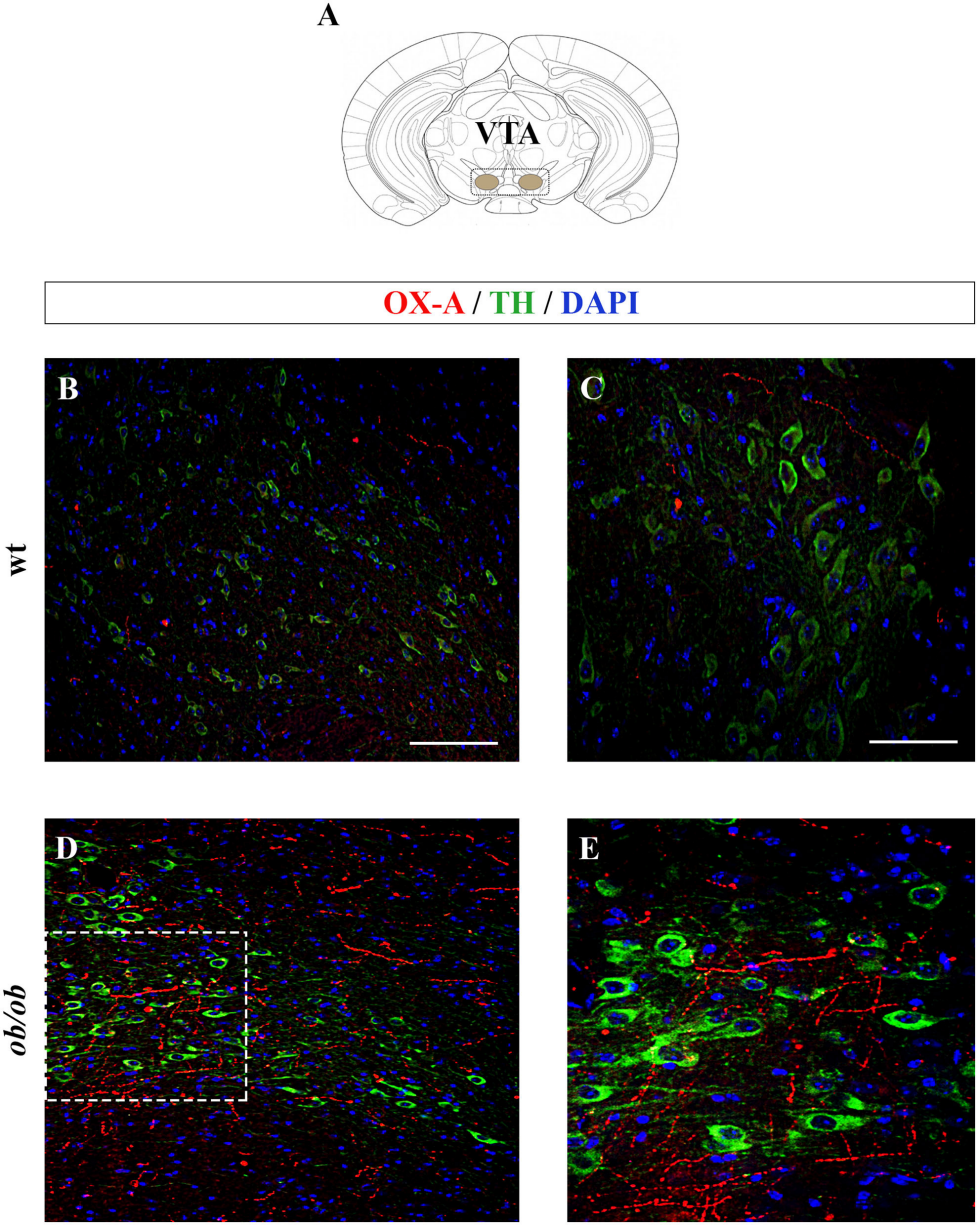
**(A)** Schematic representation of a coronal section of the mouse brain highlighting the anatomical localization of the VTA (adapted from the mouse brain atlas, (Franklin and Paxinos, 1997). **(B–E)** Confocal microscopy images showing OX-A immunolabeled axon terminals apposed to TH-expressing neurons of adult wt and *ob/ob* mice. Immunofluorescent signals are red for OX-A, green for TH, and blue for DAPI. Note the enhancement of OX-A and TH immunoreactivities in the VTA of *ob/ob* mice **(D,E)** compared to the wt mice **(B,C)**. **(E)** is a higher magnification of the boxed area in **(D)**. [Scale bar: 100 μm **(B,D)**, 50 μm (**C,E)**].

**Figure 3:**
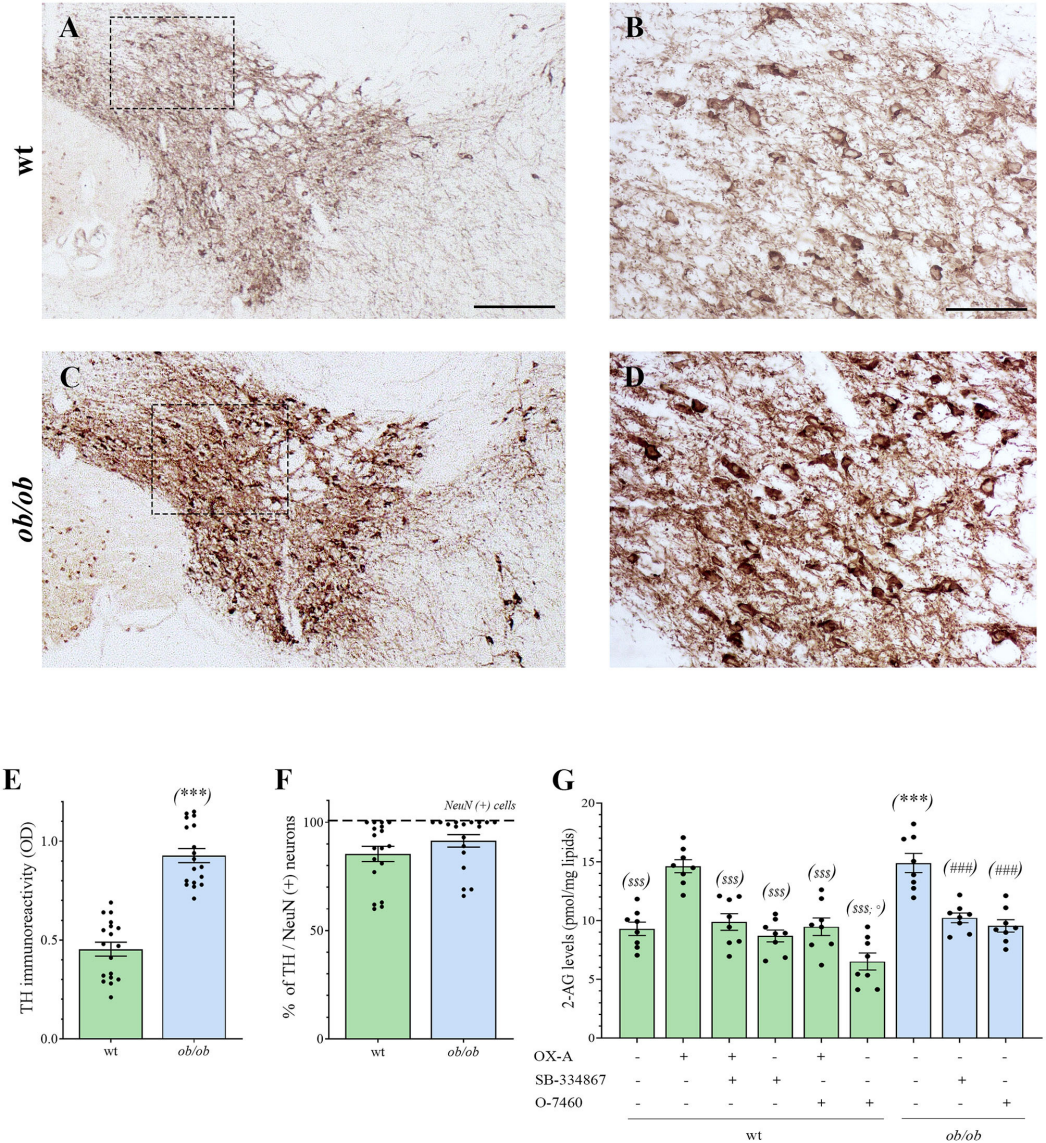
**(A–D)** Representative peroxidase-based TH staining in the VTA of wt and *ob/ob* mice. **(B,D)** are higher magnification of the boxed areas in **A** and **C**, respectively [Scale bar: 300 μm **(A,C)**, 100 μm **(B,D)**]. Note the enhancement of TH immunoperoxidase labeling in *ob/ob* mice. **(E)** Bar graph of TH peroxidase-based optical density of immunoreactivity (O.D). Data are means ± SEM from *n* ≥ 3 sections/mouse, *n* = 6 mice/genotype; ***P < 0.001 *ob/ob* vs. wt mice; two-tail *t*-test with Welch's correction. **(F)** Bar graph showing the mean percentage of TH–expressing neurons calculated as ratio vs. the number of the neuronal marker NeuN-expressing cells (TH/NeuN ratio). Data are means ± SEM from *n* ≥ 3 sections/mouse, *n* = 6 mice/genotype; *P* = 0.33; two-tail *t*-test with Welch's correction. **(G)** Levels of 2-AG in the VTA of wt (green bars) and *ob/ob* mice (blue bars). Data are from *n* = 8 mice per group and are expressed as means ± SEM; ^$$$^*P* < 0.001 wt+vehicle or wt treated vs. wt+OX-A mice; °*P* < 0.05 wt+O-7460 vs. wt+vehicle mice; ***P < 0.001 *ob/ob* vs. wt mice; ^###^*P* < 0.001 *ob/ob* treated vs. *ob/ob*+vehicle mice; one-way ANOVA followed by Bonferroni test.

Increased OX-A trafficking was found in the fibers projecting from the LH to the VTA (Figure 1A) of *ob/ob* in comparison to wt mice (Figures 1B–E), a feature confirmed by optical densitometry analysis of OX-A immunoreactivity (Figure 1F) and in line with our previous results (Cristino et al., 2013, 2016; Morello et al., 2016). DA neurons are unequivocally identified by the presence of tyrosine hydroxylase (TH), the rate-limiting enzyme of DA synthesis (Daubner et al., 2011). By confocal imaging OX-A/TH immunolabelling, we found a large distribution of OX-A-ir puncta as apposed to TH-ir neurons in the VTA of *ob/ob* compared to wt mice (Figure 2), in line with the enhancement of OX-A trafficking documented before (Figure 1F), and of TH-ir found in the vast majority of the VTA neurons by optical microscopy (Figures 3A–D). The densitometric measure of TH-ir in the VTA confirmed the enhanced expression of this rate-limiting enzyme of dopamine synthesis in *ob/ob* vs. wt mice (Figure 3E). The measure of the TH/NeuN ratio did not reveal a change in the percentage of the TH-positive neurons between *ob/ob* (92.89% ± 3.49) and wt (87.44% ± 4.23) mice through the entire volume of the VTA (Figure 3F). This result is in line with data showing that the vast majority of VTA neurons are dopaminergic (Bok et al., 2018).

Altogether, these data show that in the VTA of *ob/ob* mice an elevation of OX-A trafficking occurs concurrently with an increased expression of TH.

### The OX-A-OX1R- DAGL-α Cascade Enhances 2-AG Levels in TH-ir Neurons of *ob/ob* Mice

We and others have previously demonstrated that 2-AG can be biosynthesized upon activation of postsynaptic OX1Rs by OX-A in the periaqueductal gray area (Ho et al., 2011; Cristino et al., 2016) and arcuate nucleus of mice (Morello et al., 2016). Furthermore, 2-AG synthesis can be triggered by OX-A in DA neurons via GqPCR(OX1R)-mediated activation of the PLC-DAGL-α pathway (Tung et al., 2016). Starting from this background, we sought to investigate: (i) if enhancement of 2-AG levels occurs in the VTA of *ob/ob* vs. wt mice as an effect of increased OX-A content in this area (Figure 1F); (ii) the anatomical network which underlies the OX-A-OX1R-DAGL-α cascade in the VTA.

#### 2-AG Levels Are Increased in the VTA of Obese *ob/ob* Mice

By LC-APCI-mass spectrometric analysis of lipids extracted from the mouse VTA, we found an enhancement of 2-AG levels in *ob/ob* mice (15.04 ± 0.70 pmol/mg lipids, *n* = 6 mice per group) in comparison to wt mice (8.08 ± 0.41 pmol/mg lipids, *n* = 6 mice per group), as well as in the VTA of wt mice injected with OX-A (40 μg/kg, i.p., 2 h) (12.73 ± 0.46 pmol/mg lipids, *n* = 6 mice per group) in comparison to the wt vehicle-injected mice (8.08 ± 0.41 pmol/mg lipids). This enhancement was prevented by SB-334867 injection (30 mg/kg, i.p., 1h before OX-A treatment) (8.70 ± 0.39 pmol/mg lipids, *n* = 6 mice per group). In the VTA of SB-334867-treated *ob/ob* mice (60 mg/kg, i.p., 1 h) we found decreased levels of 2-AG (10.52 ± 0.35 pmol/mg lipids), similar to vehicle-injected wt mice (8.08 ± 0.41 pmol/mg lipids). These data demonstrate that the increase of endocannabinoid tone in the VTA is due specifically to increased OX-A signaling at OX1R. The enhancement of 2-AG levels in *ob/ob* mice and wt-OX-A injected mice was also counteracted by injection of O-7460 (12 mg/kg, i.p., 30 min), a selective inhibitor of DAGL-α (10.18 ± 0.38 pmol/mg lipids in *ob/ob* mice; 8.63 ± 0.42 pmol/mg lipids in wt-OX-A injected mice), which supports the putative activation of PLC-DAGL-α pathway downstream to the OX-A-mediated activation of OX1R (Figure 3G).

#### Anatomical and Molecular Substrates of Functional OX-A/2-AG Cross-Talk in the VTA

To study the molecular substrate of the functional cross-talk between OX-A and 2-AG at DA neurons, and its anatomical distribution in the VTA, multiple OX1R/DAGL-α/TH or OX-A/VGluT1/TH or CB1R/VGAT/TH immunofluorescence was performed in coronal sections of the VTA.

**Figure 4:**
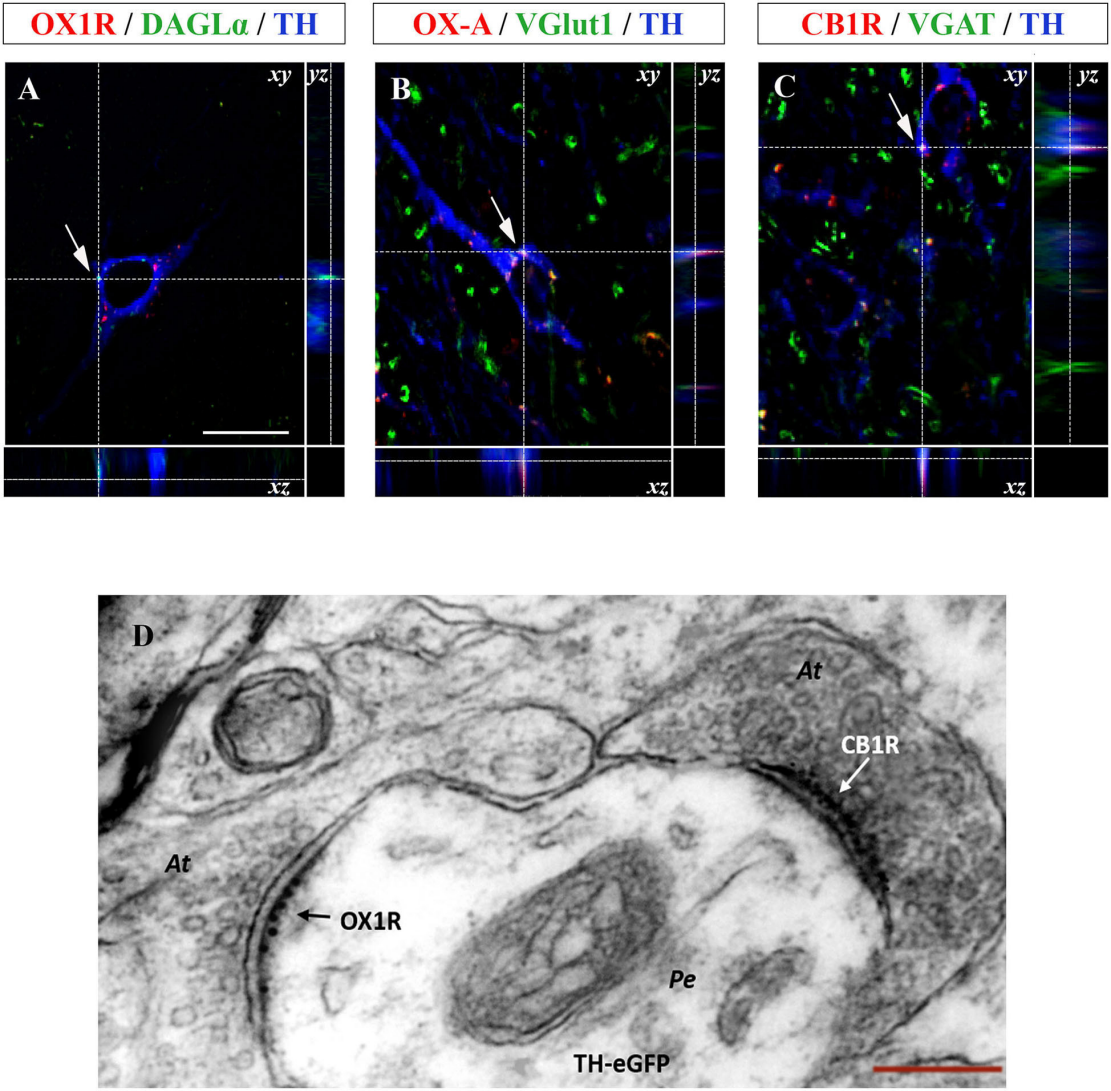
**(A–C)** Confocal microscopy images (maximum intensity projections) of three multiple immunostained sections of the VTA. **(A)** Representative image of a dopaminergic neuron showing the co-expression of OX1R (red), DAGL-α (green), and TH (blue) immunoreactivities indicated by arrows. **(B)** Representative image of a dopaminergic neuron showing OX-A/VGluT1/TH co-expression (arrow). **(C)** Representative image of a TH positive neuron (blue) receiving CB1R immunolabeled puncta (red) colocalizing with VGAT immunoreactive inputs (green). Arrows indicate the merged immunolabeling at *xy* optical intersection, which is represented by high magnification in the lateral insets showing the *yz* and *xz* intersection of the dotted lines [scale bar: 30 μm]. **(D)** Representative electron micrograph showing double OX1R/CB1R immunogold reactivity in the VTA of TH-eGFP mice. Note asymmetrical, i.e., putative excitatory, axosomatic (At) input to the perikaryon (Pe) of a TH-eGFP neuron expressing OX1R-immunogold labeling and receiving symmetrical, i.e., putative inhibitory, CB1R immunogold labeled input at a different domain of the cell membrane (right) [Scale bar: 150 nm].

Confocal analysis of OX1R/DAGL-α/TH immunolabeling revealed expression of DAGL-α, the main biosynthesizing enzyme of 2-AG, close to OX1R immunoreactivity on the plasma membrane of TH-positive neurons (Figure 4A).

Since orexinergic fibers originating from the LH and projecting to the different target areas, including the VTA, are mainly excitatory (Rosin et al., 2003), multiple TH/OX-A immunofluorescence was performed associated with detection of immunoreactivity for VGluT1, i.e., the main vesicular transporter of glutamate, as a presynaptic marker of excitatory orexinergic inputs to dopamine neurons in this area. A wide OX-A/VGluT1 co-localization was found in the fibers projecting to the vast majority of TH-positive neuronal somata (Figure 4B). Along with excitatory orexinergic inputs, DA neurons are regulated also by inhibitory inputs coming from the striatal areas (Kalivas, 1993). Therefore, the study of the anatomical pathway underlying the control of the OX-A/DA network was further extended to the inhibitory component of this circuitry by performing a multiple TH/VGAT/CB1R immunofluorescence study. In agreement with a previous study (Li et al., 2010), immunoreactivity for VGAT, i.e., the main vesicular transporter of GABA, was assumed as a presynaptic marker of inhibitory inputs to TH-expressing somata. We found inhibitory CB1R-immunolabeled GABAergic synaptic endings opposed to the somata of TH-positive neurons in the VTA (Figure 4C).

To further demonstrate the ultrastructural details of the anatomical substrate wherein orexin, endocannabinoids, and dopamine interact, we performed a CLEM study of OX1R and CB1R immunolabeling in the VTA of TH-eGFP mice. CLEM analysis revealed that TH-eGFP somata of DA neurons express OX1R labeling (10 nm immunogold particles) at postsynaptic sites of asymmetrical (i.e., putative excitatory) inputs and receive immunogold labeled CB1R-positive puncta (6 nm immunogold) at symmetrical (i.e., putative inhibitory) synapses (Figure 4D).

### OX-A Enhances Dopamine Levels in the VTA and NAc

By considering the anatomical circuitry underlying OX-A/2-AG/DA interactions in the VTA, we moved forward to investigate the functional effect of aberrant OX-A and 2-AG signaling on DA neurons, either in wt or *ob/ob* mice. This part of the study was carried out both in the VTA and NAc, the main VTA target area in the mesolimbic pathway forming the master circuitry that regulates reward-seeking behaviors and addiction (Alonso-Alonso et al., 2015).

**Figure 5:**
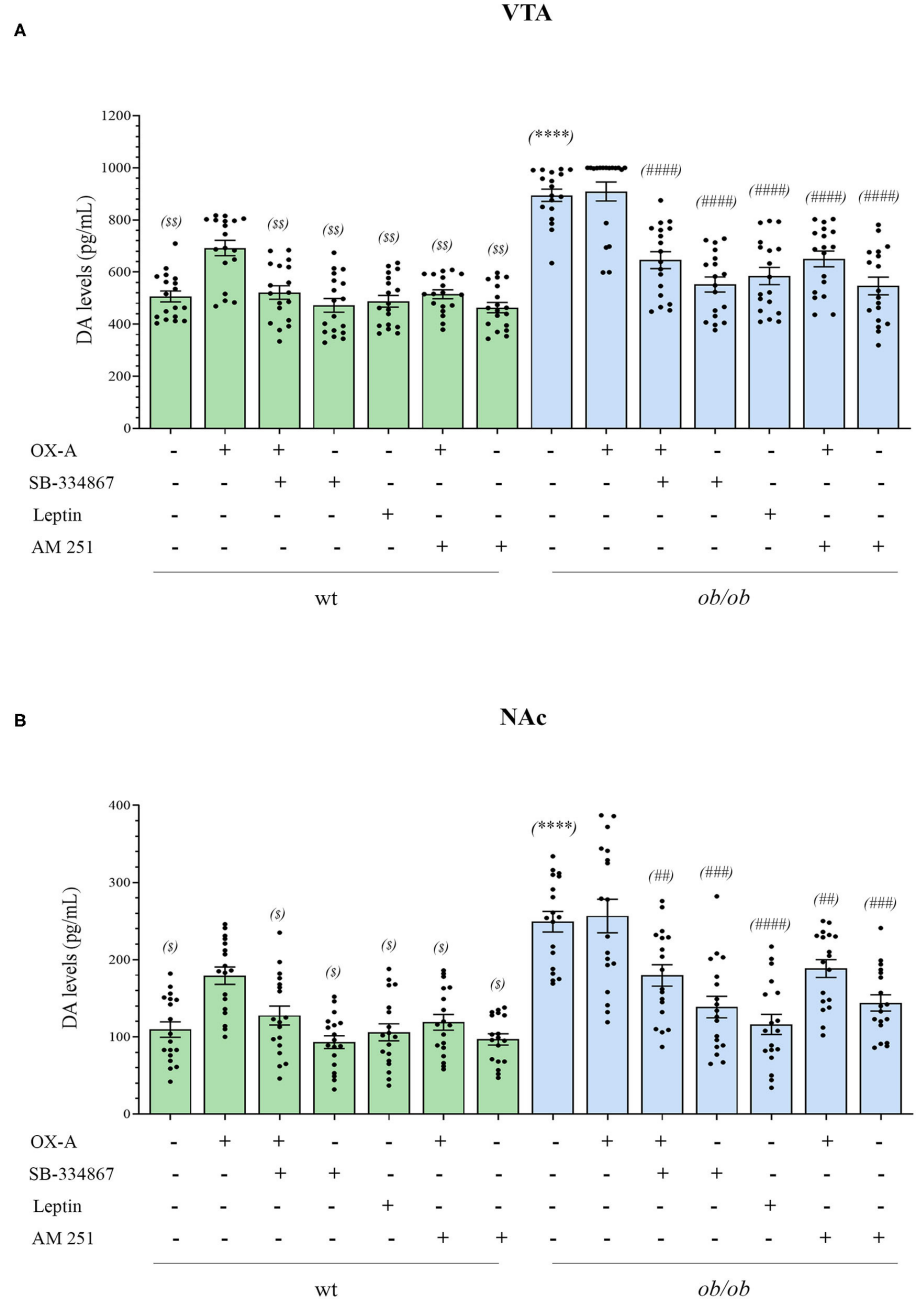
Bar graph of dopamine levels in the VTA **(A)** and NAc **(B)** of wt lean (green bars) and *ob/ob* obese (blue bars) mice. Data are means ± SEM from *n* ≥ 18 VTA or Nac sampled by punch microdissection (~1.5 mm diameter) from each brain hemisphere of a mouse, *n* = 9 mice/genotype per group; ^$^*P* < 0.05, ^$$^*P* < 0.005 wt+vehicle or wt treated vs. wt+OX-A vs. mice; ****P < 0.0001 *ob/ob* vs. wt mice; ^##^*P* < 0.005, ^###^*P* < 0.001, ^####^*P* < 0.0001 *ob/ob* treated vs. *ob/ob*+vehicle; two-way ANOVA followed by *post hoc* Tukey test.

ELISA assays revealed the enhancement of DA concentrations in the VTA and NAc of *ob/ob* mice in comparison to wt (VTA: 895.89 ± 29.12 pg/mL in *ob/ob* vs. 506.44 ± 20.79 pg/mL in wt) (NAc: 249.11 ± 13.72 pg/mL in *ob/ob* vs. 109.44 ± 10.32 pg/mL in wt) (Figures 5A,B). Such elevation was dependent on OX-A signaling since it was mimicked by OX-A injection in wt mice (40 μg/kg, i.p., 2 h) (in VTA: 692 ± 29.67 pg/mL; in NAc: 172 ± 12.66 pg/mL) in a manner attenuated by the OX1R antagonist SB-334867 (30 mg/kg, i.p., 3 h) (in VTA: 521.33 ± 25.99 pg/mL; in NAc:133.89 ± 14.50 pg/mL) and by the CB1R antagonist AM251, in wt mice (10 mg/kg, i.p., 3 h) (in VTA: 514.67 ± 16.94 pg/mL; in NAc: 118.44 ± 12.50 pg/mL). In the VTA of OX-A-injected (40 μg/kg, i.p., 2 h) *ob/ob* mice the DA levels were similar to the control *ob/ob* mice (901.11 ± 36.32 pg/mL) and, in several samples, it resulted very close to the maximum limit of detection (1,000 pg / mL) reported for the used ELISA kit. In *ob/ob* mice the DA levels were reduced by SB-334867 (60 mg/kg, i.p., 3 h) (in VTA: 552.78 ± 34.10 pg/mL; in NAc: and 138.56 ±15.90 pg/mL) or AM251 (10 mg/kg, i.p., 3 h) (in VTA: 544.11 ± 37.94 pg/mL; in NAc: 144 ± 11.74 pg/mL) injection either *per se* or before OX-A treatment [SB-334867 (60 mg/kg, i.p., 1 h) (in VTA: 645.67 ± 32.46 pg/mL; in NAc: 179.56 ± 15.30 pg/mL)] [(AM251 (10 mg/kg, i.p., 1 h) (in VTA: 651.44 ± 34.16 pg/mL; in NAc: 190.89 ± 12.06 pg/mL)]. Furthermore, DA levels were lowered in the VTA and NAc of *ob/ob* mice injected with leptin (5 mg/kg, i.p., 2 h) (in VTA: 583.22 ± 36.48 pg/mL; in NAc: 118.11 ± 17.33 pg/mL), in agreement with our previous finding concerning the inhibitory effect by this adipokine on the disinhibition of OX-A release from the LH of obese mice (Cristino et al., 2013) (Figures 5A,B).

### OX-A Signaling Drives β-Arrestin2-Mediated Desensitization of D2 Receptors in the NAc of Obese Mice

**Figure 6:**
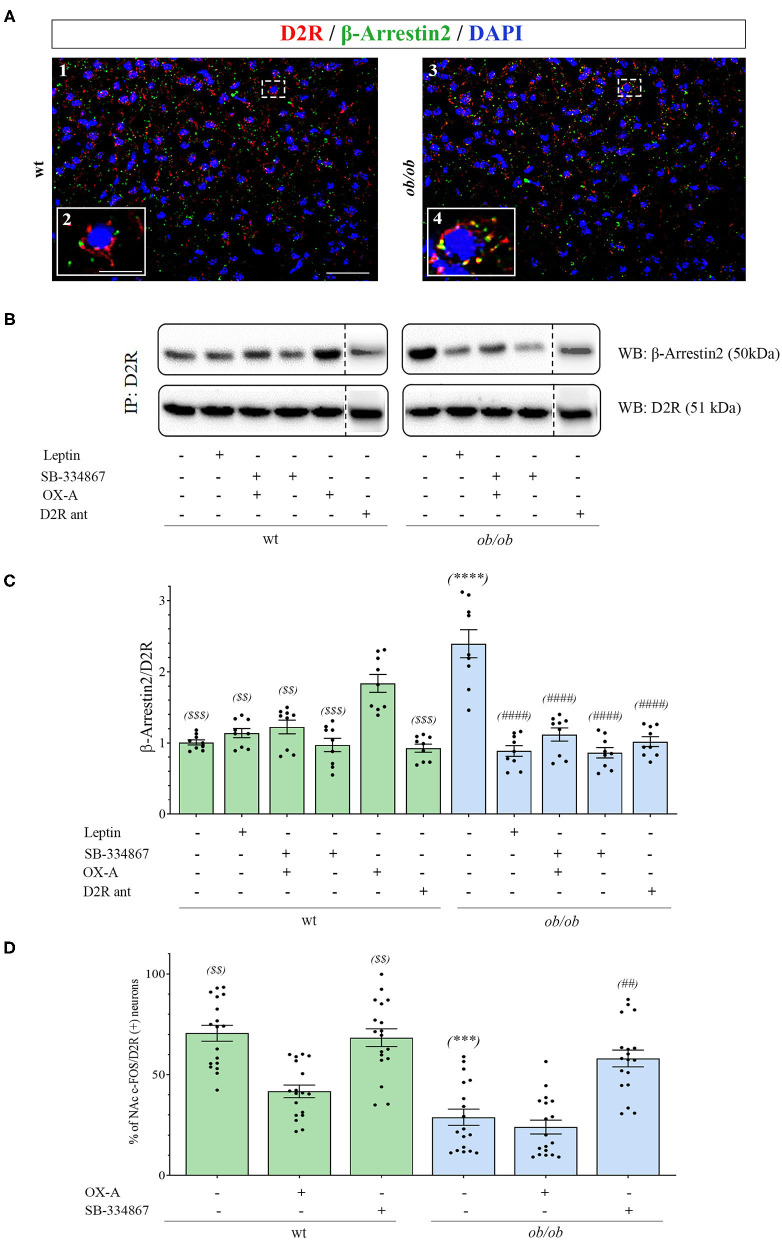
**(A)** Confocal microscopy showing D2R (red) and β-arrestin2 (green) immunoreactivities in neurons of NAc in wt (A1) and *ob/ob* mice (A3). Note the high D2/β-arrestin2 colocalization (yellow) in *ob/ob* mice in comparison to wt mainly at the plasmatic membrane as revealed by the high magnification of a representative cell in the dotted boxed area in A1 and A3 and depicted in the respective inset as A2 and A4. Images are representative of *n* ≥ 6 mice per group. [Scale bar: scale bar: 60 μm (A1 and A3), 30 μm (A2 and A4)]. **(B)** Representative immunoblots from D2R/β-arrestin2 co-immunoprecipitation assays from the NAc of wt and *ob/ob* mice. OX-A treatment (40 μg/kg, i.p., 2 h) in wt mice strongly increased the formation of the D2R/β-arrestin2 complex in comparison to vehicle-treated mice. In *ob/ob* mice the binding of D2R to β-arrestin2 was sensitive to treatment with leptin (5 mg/kg, i.p., 2 h) and SB-334867 (60 mg/kg, i.p., 3 h). **(C)** Densitometric analysis of the D2/β-arrestin2 complex. Data are means ± SEM from *n* = 8 mice/genotype per group; ^$$^*P* < 0.005, ^$$$^*P* < 0.001 wt+vehicle or wt treated vs. wt+OX-A mice; ****P < 0.0001 *ob/ob* vs. wt mice; ^####^*P* < 0.0001 *ob/ob* treated vs. *ob/ob*+vehicle mice; one-way ANOVA with Bonferroni *post hoc* **(D)** Percentage of c-*Fos***/**D2R positive neurons in the NAc of wt and *ob/ob* mice with or without SB-334867 injection (i.p., 3 h, 30 mg/kg, for wt and 60 mg/kg for *ob/ob* mice), or OX-A treatment (40 μg/kg, i.p., 2 h). Data are means ± SEM for n ≥3 sections/mouse, *n* = 6 mice/genotype per group; ^$$^*P* < 0.005 wt+vehicle or wt+SB-334867 vs. wt+OX-A mice; ***P < 0.001 *ob/ob* vs. wt mice; ^##^*P* < 0.005 *ob/ob*+SB-334867 vs. *ob/ob*+vehicle mice; two-way ANOVA followed by *post hoc* Tukey test.

To further investigate if OX-A, via 2-AG-mediated disinhibition of DA neurons in the VTA, could affect mesolimbic pathway possibly by regulating sensitivity to DA receptors in the NAc target area, we studied the immunohistochemical expression of β-arrestin2 in the NAc. Since, among the D1-D5 subtypes of DA receptors, the D2 subtype is mainly involved in reward processing of drugs and natural stimuli including food (Zlomuzica et al., 2018), we analyzed β-arrestin2 expression in D2R-positive neurons by performing β-arrestin2/D2R co-immunostaining and co-immunoprecipitation assays in the NAc of wt and obese mice. By confocal microscopy analysis of D2R/β-arrestin2 immunosignal we found that, contrary to wt mice (Figure 6A_1_), a wide colocalization was found in the soma of NAc neurons of *ob/ob* mice (Figure 6A_3_) wherein merged yellow signals were more frequently observed (see in Figure 6 the comparison between inset A_2_ and A_4_ as a high magnification of the cell in the dotted boxed area in A_1_ and A_3_, respectively). These data suggest a pivotal role of OX-A in the overactivation, and subsequent desensitization, of DA signaling at the mesolimbic circuitry, which could underlie subsequent overeating and food-addictive behaviors. In agreement with immunohistochemical data, the β-arrestin2/D2R co-immunoprecipitation assay showed a significant increase of β-arrestin2/D2R ratio in the co-immunoprecipitate complex from the NAc of *ob/ob* vs. wt mice. OX-A injection in wt lean mice (40 μg/kg, i.p., 2 h) enhanced the coupling of β-arrestin2 to D2R, similar to what observed in *ob/ob* mice (1.83 ± 0.15 for wt + OX-A vs. 2.40 ± 0.21 for *ob/ob* mice) (Figures 6B,C). On the contrary, SB-334867 injection prevented, or strongly reduced, the formation of the β-arrestin2/D2R complex in wt (SB-334867, 30 mg/kg, i.p., 3 h) (0.97 ± 0.11) and wt OX-A injected mice (SB-334867, 30 mg/kg, i.p, 1h, followed by OX-A injection: 40 μg/kg, i.p., 2 h) (1.22 ± 0.12), as well as in *ob/ob* mice (SB-334867: 60 mg/kg, i.p., 3 h) (0.86 ± 0.09), thus confirming the involvement of OX-A and OX1R in β-arrestin2-mediated D2R desensitization. In the same way, obese *ob/ob* mice injected with leptin (5 mg/kg, i.p., 2 h) or with the selective D2R antagonist L741 (1.5 mg/Kg, i.p. 30 min) showed a lower β-arrestin2/D2R ratio as compared to the basal condition of vehicle-injected *ob/ob* mice (0.85 ± 0.09 for *ob/ob* + leptin and 1.02 ± 0.07 for *ob/ob* + D2R antagonist vs. 2.40 ± 0.21 for *ob/ob* mice), and similar to leptin- or D2R antagonist-injected wt mice (1.14 ± 0.08 for wt + leptin mice and 0.93 ± 0.06 for wt + D2R antagonist-treated mice) (Figures 6B,C). In agreement with the abundant formation of the β-arrestin2/D2R complex in *ob/ob* mice, as revealed by the co-immunoprecipitation assay, a reduced percentage of c*-Fos***/**D2R positive neurons was found in the NAc of *ob/ob* (29 ± 5.58%) in comparison to wt mice. This reduction was mimicked by OX-A injection in wt mice (40 μg/kg, i.p., 2 h) (40 ± 4.12%) and counteracted by SB-334867 injection in both wt (30 mg/kg, i.p., 3 h) (68.13 ± 5.21%) and *ob/ob* mice (60 mg/kg, i.p., 3 h) (55 ± 6.31%) (Figure 6D). Consistent with the immunohistochemical and molecular findings (Figures 6A,B) which identify the D2R as the main target of DA signaling in the NAc, the pharmacological blockade of D2R by the administration of selective D2R antagonist L741 (1.5 mg/kg, i.p. 30 min) prevented D2R desensitization in obese mice by reducing the β-arrestin2/D2R co-immunoprecipitation ratio vs. that observed in vehicle-injected *ob/ob* mice (Figure 6C).

In summary, in this part of the study we found that:

1. D2R represents the main target of DA signaling in the NAc. OX-A-mediated enhancement of DA signaling in obese mice eventually reduced D2R activity by promoting the formation of D2R/β-arrestin2 complex and D2R desensitization.
2. Almost half of the D2R-positive neurons in the NAc of *ob/ob* mice was constitutively insensitive to DA signaling as revealed by c-Fos immunoreactivity. This finding was reproduced by OX-A administration to wt mice and prevented by administration of the selective D2R antagonist L741, or by the administration of OX1R antagonist SB334867, *per se*, in obese mice, or before OX-A injection, in wt mice.

Both these findings suggest that the motivational aspects of reward processing mediated by D2R are altered in obese mice.

## Discussion

Because OX-A and 2-AG have been implicated in addiction-related behaviors (España, 2012; Oleson and Cheer, 2012; Baimel et al., 2015; Hernandez and Cheer, 2015), and OX-A triggers 2-AG biosynthesis via the PLC/DAGL-α pathway downstream to OX1R, we were interested in identifying the neural circuit underlying the OX-A/2-AG interplay in the VTA, and in testing if OX-A alters DA levels in the VTA by modulating local 2-AG synthesis and release in mice. With this aim we studied wt lean mice and leptin knockout obese *ob/ob* mice, this latter being characterized by an elevated basal tone of OX-A signaling due to the lack of leptin signaling (Cristino et al., 2013).

Despite the significant orexinergic innervation of the VTA, where both OX1R and OX2R receptors are found in neurons of the mesolimbic pathway (Marcus et al., 2001; Narita et al., 2006), OX-A/OX1R signaling represents the master player of dopaminergic neurons. Several studies have demonstrated that OX-A infusions into the VTA can increase DA concentration in the shell (Vittoz et al., 2008), core (España et al., 2011) or both, of the Nac (Narita et al., 2006). Moreover, an increase of local DA release in the NAc shell has been demonstrated following OX-A treatment of brain slices (Patyal et al., 2012). We report that 2 h following i.p. OX-A injection in mice can increase DA levels in the VTA and NAc in concomitance with the enhancement of 2-AG levels and via the potential CB1R-mediated disinhibition of GABAergic inputs to TH-ir neurons. OX-A-induced elevation of DA was prevented by the OX1R antagonist SB-334867 or by the CB1 antagonist AM251 injected i.p. 1 h before OX-A. In addition to glutamatergic signaling contributing to cocaine-induced sensitization (Borgland et al., 2006), GABAergic transmission is also an important modulator of VTA dopaminergic activity. OX-A application to VTA slices, in addition to increasing the firing of dopaminergic neurons, also directly decreased the firing of GABAergic neurons, suggesting the existence of another cellular mechanism related to OX-A modulation of GABAergic activity triggered by endocannabinoid signaling at the VTA and increasing VTA dopaminergic activity by retrograde disinhibition via CB1R inhibition of GABAergic inputs (Alger, 2002; Melis et al., 2004; Ho et al., 2011).

Electrophysiological studies further demonstrated that activation of OX1R in VTA dopaminergic neurons initiates a Gq/11-coupled PLC-DAGL pathway leading to the biosynthesis of 2-AG, which, retrogradely, activates CB1R at both inhibitory and excitatory inputs to DA neurons and regulating burst firing and DA release of VTA-DA neurons (Tung et al., 2016). In resting conditions, around 50% of DA neurons in this area are innervated by inhibitory GABAergic inputs (Grace and Bunney, 1984). Despite the fact CB1R was expressed at both GABAergic and glutamatergic inputs to VTA dopaminergic neurons (Mátyás et al., 2008), those innervated by OX-A afferences are mostly synaptically contacted by GABAergic CB1R expressing inputs (Tung et al., 2016). In line with this functional mechanism, we here provide analytical quantification for 2-AG levels in the VTA and show that OX-A/OX1R-mediated activation of the PLC-DAGLα pathway underlies enhanced 2-AG biosynthesis in the VTA of both OX-A-injected lean wt mice and obese *ob/ob* mice. In fact, this effect was prevented by SB334867 injection or by inhibition of DAGLα with 0-7460. Furthermore, confocal and electron microscopy analysis revealed the cytoarchitectonic distribution of the main enzymes, receptors, and neurotransmitters forming the OX-A-controlled DA network in the mouse VTA (Graphical abstract, A and B).

Consistent with the enhancement of OX-A trafficking from the LH to different target areas in the brain in *ob/ob* mice (Cristino et al., 2013; Morello et al., 2016), here we found intense OX-A immunoreactivity in the fibers projecting to the VTA and NAc. In both lean OX-A-injected and obese *ob/ob* mice, the elevation of OX-A signaling in the VTA was accompanied by enhancement of 2-AG and DA levels in the VTA and NAc. All these effects were prevented by OX1R antagonism and are in line with the reduced DA levels and release observed previously in *Hcrt*-KO mice (Shaw et al., 2017). From the neurobiological point of view, the aberrant disinhibition of OX-A neurons described in *ob/ob* mice (Cristino et al., 2013; Becker et al., 2017) could explain the enhancement of orexinergic tone associated with the pathological hyperarousal typical of compulsive and addictive behaviors (Boutrel et al., 2005).

Several studies report that such behaviors, with either food or drugs of abuse, drive dopaminergic signaling via D2R, thereby inducing a paradoxical impairment in reward processes and to subsequent tolerance and addictive behavior (Wang et al., 2002; Koob and Le Moal, 2005; Borgland et al., 2006; Avena et al., 2008; Johnson and Kenny, 2010). Our results reveal that D2R undergoes β-arrestin2-mediated desensitization in the NAc upon longer-lasting activation induced by prolonged exposure to DA in obese mice (Graphical abstract, C). This condition is reflected by the reduction of c-*Fos* expression, a marker of neuronal functional activity, in D2R neurons. Both the D2R molecular internalization by the β-arrestin2 complex, and c-Fos reduction were prevented by injection of the selective D2R antagonist L741, as well as by SB334867, in agreement with similar results obtained in high fat fed obese mice (Valdivia et al., 2014).

Our immunohistochemical data reporting the enhancement of TH-ir in VTA neurons of *ob/ob* mice are, apparently, not in line with those referring to a reduction or no difference between *ob/ob and* wt mice (Fulton et al., 2006). This discrepancy could be possibly ascribed to a different method of TH quantification, which we based on single-cell optical imaging densitometry, hence avoiding to measure the tissue outside TH-ir perikarya. Additionally, the way and duration of the pharmacological treatments and the method for DA quantification were also different in our study, wherein leptin i.p. injection reduced DA levels in the VTA and NAc, in contrast to the DA elevation observed in leptin i.c.v.-injected *ob/ob* mice (Leinninger et al., 2009).

Several anatomical and functional interactions occur between the orexinergic and dopaminergic systems: (i) dendrites and somata of DA neurons receive OX-A-positive afferences, as demonstrated in this work and by others (Fadel and Deutch, 2002); (ii) OX-A increases the firing rate of TH-positive neurons in the VTA (Tung et al., 2016); (iii) OX-A increases the firing of VTA DA neurons projecting to the shell region of the NAc (Baimel et al., 2017); (iv) blocking D2R reduces hyperlocomotion and stereotypy induced by i.c.v. injection of OX-A (Nakamura et al., 2000). OX-A and drugs such as amphetamines share the ability to induce hyperlocomotion, grooming, and stereotypy in rats, typically considered behaviors of enhanced dopaminergic tone (Ida et al., 1999). These effects are inhibited by antagonism of D2R, thereby suggesting the involvement of the dopaminergic system in behavioral responses induced by OX-A (Nakamura et al., 2000). Accordingly, clinical observations have reported that human patients suffering from narcolepsy, due to almost complete loss of orexinergic neurons, despite long-term medical treatment with amphetamines and other wakefulness-stimulating substances, rarely develop an addiction to stimulant drugs of abuse (Kilduff and Peyron, 2000; Nishino et al., 2000; Barateau et al., 2016). In line with these clinical data in humans, orexin deficient mice (knock-out for *Hcrt*) show a lower consumption of sucrose when available *ad libitum* compared to littermate wt mice (Matsuo et al., 2011). Orexinergic neurons are activated by physiological homeostatic stimuli, including hypoglycemia and caloric restriction (Milbank and Lopez, 2019). Moreover, they are activated also in response to environmental cues like food, even during periods of relatively high energy abundance, thus indicating the ability of the orexinergic system to be activated in response to anticipation of rewards induced by high palatable food (Choi et al., 2010). This latter activates orexinergic neurons that project to the VTA by increasing motivational behavior that drives the craving for appetitive reinforcers (Valdivia et al., 2014). Likewise, the anticipation of preferred, palatable food and/or its consumption, with respect to a non-preferred food, also increases circulating 2-AG levels in human volunteers, and this response is accentuated in obese individuals and patients with binge eating disorder (Monteleone et al., 2016, 2018), in agreement with the enhanced OX-A/2-AG interaction that we have found here to occur in the VTA of *ob/ob* mice. Additionally, retrograde endocannabinoid signaling at CB1 receptors in the VTA is a well-established mechanism for the reinforcement of DA signaling in this area and of its behavioral consequences in food reward (Bacharach et al., 2018; Wenzel and Cheer, 2018). Altogether, our data suggest that aberrant OX-A signaling triggers a vicious OX1R/PLC-DAGL/2-AG/CB1 -mediated loop which promotes DA production and, possibly, release in the VTA and NAc. Excessive DA levels then act mainly via D2R in the mesolimbic area and cause D2R desensitization through the beta-arrestin2 pathway, with subsequent impairment of the rewarding process. These data put forward novel CB1R-mediated mechanisms for the regulatory action of OX-A in the mesolimbic dopaminergic system.

## Data Availability Statement

The raw data supporting the conclusions of this article will be made available by the authors, without undue reservation.

## Ethics Statement

The animal study was reviewed and approved by European Communities Council Directive of September 22, 2010 (2010/63/EU) and the Italian Decree n.26/2014, authorization n. 152/2020-PR.

## Author Contributions

LC conceived and designed the study. PG, VD, and LC contributed to the theoretical framework. VD and LC wrote the manuscript. LT prepared the figures. LT, LD'A, AF-R, and RI performed immunohistochemical studies. LT and RI performed electron microscopy study. AF-R and NF performed co-immunoprecipitation assay. LT, LD'A, and AF-R performed ELISA assay and FP performed LC-MS analysis. All the authors analyzed the data. All authors read and approved the final manuscript.

## Conflict of Interest

The authors declare that the research was conducted in the absence of any commercial or financial relationships that could be construed as a potential conflict of interest.
